# Neural Correlate of Transition Violation and Deviance Detection in the Songbird Auditory Forebrain

**DOI:** 10.3389/fnsys.2018.00046

**Published:** 2018-10-09

**Authors:** Mingwen Dong, David S. Vicario

**Affiliations:** ^1^Behavior and Systems Neuroscience, Psychology Department, Rutgers, the State University of New Jersey, New Brunswick, NJ, United States; ^2^Behavior and Systems Neuroscience, Psychology Department, Rutgers, the State University of New Jersey, New Brunswick, NJ, United States

**Keywords:** sequence sensitivity, transition probability, predictive coding, mismatch negativity, stimulus-specific adaptation, context-dependent processing

## Abstract

Deviants are stimuli that violate one's prediction about the incoming stimuli. Studying deviance detection helps us understand how nervous system learns temporal patterns between stimuli and forms prediction about the future. Detecting deviant stimuli is also critical for animals' survival in the natural environment filled with complex sounds and patterns. Using natural songbird vocalizations as stimuli, we recorded multi-unit and single-unit activity from the zebra finch auditory forebrain while presenting rare repeated stimuli after regular alternating stimuli (alternating oddball experiment) or rare deviant among multiple different common stimuli (context oddball experiment). The alternating oddball experiment showed that neurons were sensitive to rare repetitions in regular alternations. In the absence of expectation, repetition suppresses neural responses to the 2nd stimulus in the repetition. When repetition violates expectation, neural responses to the 2nd stimulus in the repetition were stronger than expected. The context oddball experiment showed that a stimulus elicits stronger neural responses when it is presented infrequently as a deviant among multiple common stimuli. As the acoustic differences between deviant and common stimuli increase, the response enhancement also increases. These results together showed that neural encoding of a stimulus depends not only on the acoustic features of the stimulus but also on the preceding stimuli and the transition patterns between them. These results also imply that the classical oddball effect may result from a combination of repetition suppression and deviance enhancement. Classification analyses showed that the difficulties in decoding the stimulus responsible for the neural responses differed for deviants in different experimental conditions. These findings suggest that learning transition patterns and detecting deviants in natural sequences may depend on a hierarchy of neural mechanisms, which may be involved in more complex forms of auditory processing that depend on the transition patterns between stimuli, such as speech processing.

## 1. Introduction

Sounds and their sequence in the natural environment are variable but often occur in patterns. Learning these patterns is adaptive because they can be used to predict future stimuli and facilitate the detection of deviant stimuli which may indicate novelty or danger. Studying deviance detection can help us understand how sensory system learns temporal patterns between stimuli and forms predictions about the future stimuli. It also provides a test for the predictive coding hypothesis, which states that sensory system constantly makes predictions about future stimuli to reduce uncertainty about the environment (Friston and Kiebel, 2009; Friston, 2010). In the auditory system, deviance detection has been widely studied using the oddball paradigm, in which one stimulus (oddball) is presented rarely in sequences of another common stimulus (standard). Using pure tone stimuli that differed in acoustic frequency, Ulanovsky et al. (2003) found that the oddball tone elicited stronger neural responses than the standard tone in the auditory cortex of anesthetized cats. This is the so-called oddball effect and the suggested neural mechanism was stimulus-specific adaptation (SSA, reduction in neural responses to repeated stimuli). Later, SSA has been found in the subcortical nonlemniscal regions of inferior colliculus (IC) and medial geniculate body (MGB) (Antunes et al., 2010; Antunes and Malmierca, 2011; Nieto-Diego and Malmierca, 2016). Similar phenomena have also been found in other species like songbirds (Beckers and Gahr, 2012; Hershenhoren et al., 2014; Malmierca et al., 2015). However, deviance detection is not limited to these simple stimuli and sequences. Deviant sounds in the natural environment occur in more complex patterns. For example, the stimulus pattern can be the alternation of two sounds instead of the repetition of just one, there can be multiple different standard stimuli instead of only one, and the sounds can often be more complex than pure tones. Indeed, mismatch negativity (MMN), a physiological correlate of deviance detection in human EEG studies, has been observed not only in the simple oddball paradigm (Näätänen et al., 2007) but also in many more complex situations (Paavilainen, 2013). For example, rare repetitions in a sequence of alternating stimuli elicited MMN (Nordby et al., 1988; Cornella et al., 2012); in another case, MMN was seen in the oddball paradigm even when two stimuli had similar acoustic structures but belonged to different phonetic categories (Sharma and Dorman, 2000; Silva et al., 2017). However, at the neural level, there have not been as many experiments studying deviance detection at the same complexity as in MMN.

To explore whether auditory neurons are sensitive to the transition patterns between stimuli and detect deviants in complex situations, we conducted two experiments: (1) alternating oddball experiment, (2) context oddball experiment. In both experiments, zebra finches were used as the animal model and we recorded multi-unit and single-unit activity from the auditory forebrain. In the alternating oddball experiment, stimulus repetitions were infrequently presented in ongoing sequences of two stimuli that were presented in either alternating (repetitions were rare & unexpected) or random order (repetitions were common). We hypothesized that neurons would respond similarly to the two stimuli in the repetition when repetitions are common (random condition), but differently when repetitions are rare (alternating condition). By comparing the neural responses to the two stimuli in the alternating and random conditions, we tested whether auditory neurons were sensitive to the transition probabilities between stimuli set up by the alternation. In the context oddball experiment, one target stimulus was presented among several different context stimuli. The context stimuli always shared acoustic similarities with each other. The target stimulus is standard when it is similar to the context stimuli but deviant when it is different from the context stimuli (differed in familiarity or acoustic categories). We hypothesized that the target stimulus would elicit stronger neural responses when it is deviant than when it is standard. By comparing the neural responses when the target stimulus was deviant vs. when it was standard, we tested whether auditory neurons can detect deviants when there are multiple context stimuli (standards in the classical oddball paradigm). Responses were assessed both using the typical encoding analysis methods, and from a decoding perspective that may be relevant to understanding neural processing along the auditory pathway. The results from these experimental approaches demonstrated neural sensitivity to sound sequences and context, phenomena that cannot be understood from a simple SSA or synaptic habituation perspective, in paradigms that are relevant to the kinds of deviants that engage mismatch negativities (MMN) (Garrido et al., 2009) in human studies. This implies that the auditory system could learn transition patterns between stimuli and constantly make predictions about the future stimuli, consistent with the predictive coding framework (Friston and Kiebel, 2009).

## 2. Materials and methods

### 2.1. Subjects

This study used 14 adult (>130 days) male zebra finches (8 for the alternating oddball experiment and 6 for the context oddball experiment, see below). All birds were housed in a general aviary with other zebra finches at Rutgers University under a 12 h:12 h light/dark cycle and provided with water and food *ad libitum*. All experimental procedures were approved by the Institutional Animal Care and Use Committee of Rutgers University.

### 2.2. Surgery

Birds were prepared for electrophysiological recording under isoflurane anesthesia (1–2% in oxygen). The anesthetized bird was placed in a stereotaxic device, feathers on the scalp were removed and 0.04 cc Marcaine (0.25%) was injected under the scalp. Then, a midline horizontal incision was made and enlarged to expose the skull. The outer layer of the skull was removed over the region of interest around the bifurcation of the mid-sagittal sinus. Dental cement was then used to form a small round chamber over the opening, and a metal pin was attached to the skull to keep the bird's head fixed during subsequent awake electrophysiological recording. The bird received an injection of 0.04 cc Metacam (5 mg/mL) for post-operative analgesia and was closely monitored for recovery.

### 2.3. Electrophysiological recording

**Figure 1:**
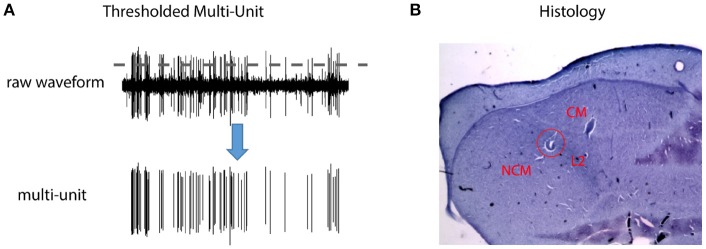
Thresholded multi-unit activity (MUA) and anatomical locations of the recording sites. **(A)** Multi-unit spike trains were obtained by thresholding the raw waveforms (2.5 standard deviation above the mean). **(B)** Cresyl Violet staining of a sagittal section at lateral 0.75 mm from the songbird auditory forebrain. Dorsal is up and caudal is to the left. The red circle marks a lesion in field L2, which has a characteristically darker color than the surrounding areas due to closely packed neurons. The area caudal to field L2 includes Caudomedial Nidopallium (NCM, bottom left), and the area dorso-rostral is Caudal Mesopallium (CM). The anatomical locations of the recording sites were reconstructed with respect to position of the lesions in individual animals. Because the three structures did not show any systematic differences in the SI or decoding results, the data from them were pooled and reported together.

After 2 days of recovery, the bird was restrained in a custom tube, and fixed to the stereotaxic frame by clamping the previously implanted pin. Then, a small craniotomy exposed the dura over the recording area. Sixteen electrodes (Type ESI2ec, impedance: 2–3 MOhms, Thomas Recording) were lowered into the auditory forebrain (1 mm lateral from midline, 1.5 mm rostral to Y-point) of the two hemispheres (8 electrodes per hemisphere). Figure 1 shows the three main structures of the auditory forebrain: field L2, Caudomedial Nidopallium (NCM), and Caudal Mesopallium (CM). Field L2 is analogous to the primary auditory cortex in mammals, and NCM and CM are similar to the superficial layer of the primary auditory cortex or the secondary auditory cortex in mammals (Brainard and Doupe, 2013). A speaker placed 30 cm in front of the bird was used for stimulus presentation. All stimuli were equated for RMS amplitude, with peak amplitude of 65 dB SPL (A scale). White noise shaped with the amplitude envelope of zebra finch song was then used to search for responsive sites. Once the electrodes showed auditory-evoked activity characteristic of the target area, playback of experimental stimuli began. A power 1401 (CED, Cambridge, England) was used for both stimulus presentation and neural recording. Neural activity was amplified (x 19,000), filtered (0.5–5 kHz bandpass), digitized (25 kHZ), and stored for further analysis.

Multi-unit activity (MUA) was obtained by thresholding the raw waveforms (2.5 standard deviation above the mean) for each electrode (Figure 1). Single unit activity (SUA) was discriminated by feeding the raw waveforms into the automatic spike sorting algorithm *waveclus* described by Quiroga et al. (2004). Sorted single units were included in the analysis only if the percentage of inter-spike intervals less than 2 ms (contamination rate) was less than 2%. For each electrode/unit, neural response to each stimulus trial was computed by subtracting the average firing rate during the baseline period (1/4 of the inter-stimulus interval before the stimulus) from the firing rate during the stimulus period (plus 10% of stimulus duration).

### 2.4. Auditory stimuli

**Figure 2:**
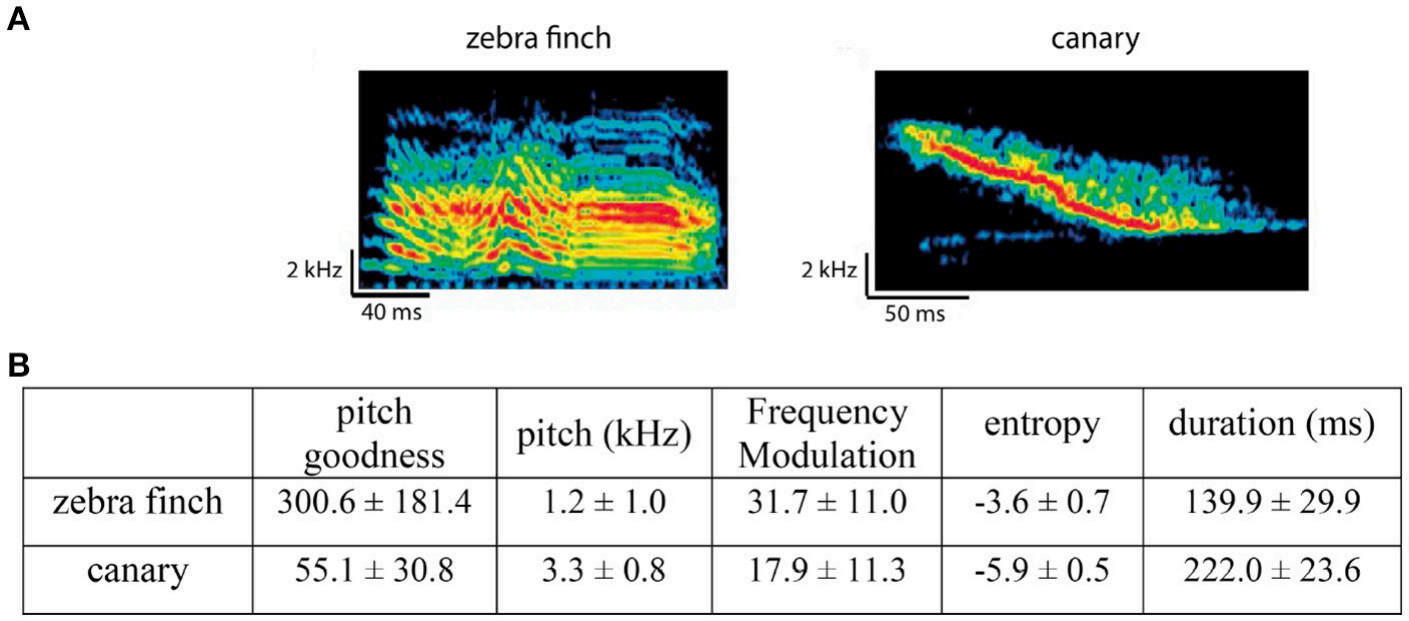
Comparison between zebra finch and canary song syllables. **(A)** Example spectrograms of one zebra finch syllable and one canary syllable. **(B)** Major acoustic differences between zebra finch and canary syllables, measured using Sound Analysis Pro (Tchernichovski et al., 2000). Note that the difference in duration is from the stimuli used in the context oddball experiment. The zebra finch and canary syllables used in the alternating oddball experiment were chosen to have the same duration (within each stimulus set).

The stimuli were syllables from zebra finch songs (recordings from our lab) and canary songs (recordings from our lab and on-line resources). Zebra finch and canary syllables had different acoustic features (measured using Sound Analysis Pro from Tchernichovski et al., 2000) and potentially belonged to different categories. Figure 2 shows examples of zebra finch and canary syllables and their major acoustic differences (e.g., frequency modulation, pitch, entropy, …).

### 2.5. Alternating oddball experiment

**Figure 3:**
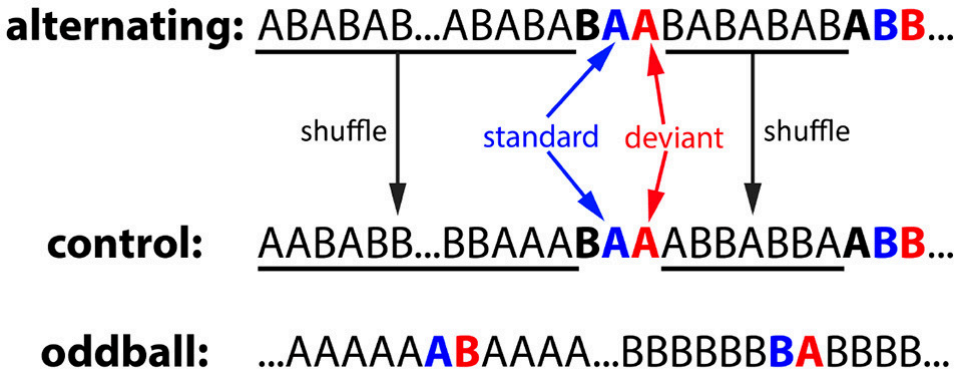
Experiment 1: alternating oddball experiment and the stimulus sequences used in the control, alternating, and oddball conditions. In the alternating condition, two stimuli were initially presented in alternation for 25 times to familiarize the bird with the stimuli and the alternation pattern. Then, rare repetitions were presented after a variable 4–10 regular alternations. The deviant (2nd stimulus in the repetition), standard (1st stimulus in the repetition), and the stimulus immediately before them formed a “triplet.” In the control condition, the number of stimulus trials and the positions of the triplets were the same as those in the alternating condition, however, stimulus sequences between the triplets were shuffled (not in alternation). In the oddball condition, two stimuli were presented in two blocks with different probabilities. For notation purposes, when a stimulus is presented with low probability, it is called the deviant and the stimulus immediately preceding it is called the standard. Deviant and standard are color-coded with red and blue, respectively.

The alternating oddball experiment included 3 conditions: alternating, control, and oddball (Figure 3). Each condition used two stimuli of the same duration: a zebra finch syllable and a canary syllable. In all three conditions, the inter-stimulus interval (ISI, onset-to-onset) was 1 second and the stimulus duration ranged from 140 to 190 ms. The particular stimuli used for the different conditions were counterbalanced across birds. In the alternating condition, the two stimuli were first presented in an alternating order for 25 times (…ABABAB…), then rare repetitions of one of the two stimuli (AA or BB) were presented after a variable 4–10 common alternations (…ABABABA**BAA**BAB…). In total, there were 20 AA repetitions and 20 BB repetitions. For the repeated pairs, the 2nd stimulus was called the deviant because it violated the alternating regularities from the preceding sequence, while the 1st stimulus was called the standard. In the control condition, the stimulus sequence was generated from the alternating condition: the deviant, standard, and the stimulus immediately before it were kept at the same position as a triplet whereas the stimulus sequence between the triplets were shuffled. Consequently, the 2nd stimulus in the repetitions was deviant in the alternating condition but not in the control condition (still called deviant for notation purposes). The typical oddball condition were also included for comparison purposes. Two stimuli (A & B) were presented in two blocks. In the 1st block, stimulus A was presented after a variable 4–10 repetitions of stimulus B. In the 2nd block, the role of the two stimuli were reversed. For notation purposes, the rare stimulus was called the deviant and the stimulus immediately preceding it was called the standard.

### 2.6. Context oddball experiment

**Figure 4:**
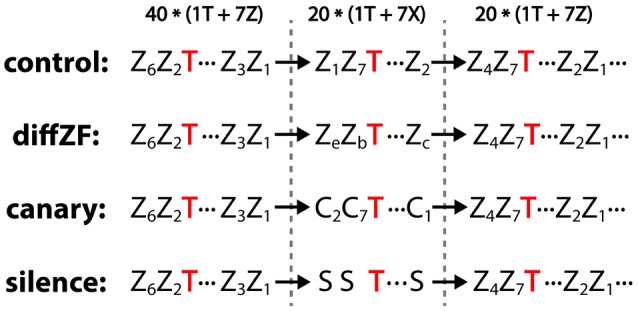
Experiment 2: context oddball experiment and the stimulus sequences used in the control, diffZF, canary, and silence conditions. In all conditions, stimuli were presented in 3 consecutive blocks. In the 1st block, one target stimulus (T) was presented with 7 different zebra finch syllables (Z) for 40 repetitions each in a shuffled order. In the 3rd block, the same stimuli as in the 1st block were presented for 20 repetitions each in a shuffled order. In the 2nd block, the same target stimulus was presented among 7 context stimuli (indicated as X), which differed depending on the experimental conditions. In the control condition, the context stimuli were the same zebra finch syllables as those in the 1st and 3rd block. In the diffZF condition, the context stimuli were 7 novel zebra finch syllables that were different from those in the 1st and 3rd block. In the canary condition, the context stimuli were 7 syllables from the canary songs. In the silence condition, the context stimuli were silence intervals instead of physical sounds. Note that the target stimulus occurs with the same probability across blocks in all but silence conditions. Subscripts are used to differentiate different stimuli of the same acoustic category.

The context oddball experiment included 4 conditions: control, diffZF, canary, and silence (Figure 4). In each condition, stimuli were presented in 3 consecutive blocks. In the 1st block, one target stimulus and 7 context stimuli were played for 40 repetitions each in a shuffled order. In the 2nd block, the same target stimulus was presented but with 7 different context stimuli for 20 repetitions each in a shuffled order. In the 3rd block, the same stimuli as in the 1st block were presented in a shuffled order but only for 20 repetitions. The 1st block sets up a baseline context in which the target stimulus is normally played. The 2nd presents a new set of context stimuli, which potentially contrast with the target stimulus and make the target a deviant. The 3rd block controls for potential unknown changes in neural responses over time.

In all conditions, the target stimuli were syllables from zebra finch songs and the context stimuli in the 1st and 3rd block were also zebra finch syllables. However, the context stimuli in the 2nd block were different in different experimental conditions. In the control condition, the context stimuli in the 2nd block were the same as those in the 1st and 3rd block. In the diffZF condition, the context stimuli in the 2nd block were also zebra finch syllables, however, they were different from those in the 1st and 3rd block and novel to the birds. In the canary condition, the context stimuli in the 2nd block were canary song syllables. They were from a different species and novel to the birds. In the silence condition, the context stimuli in the 2nd block were actually silence intervals instead of physical sounds. In the control, diffZF, and canary conditions, the ISI was always 1.2 s and there were no extra gaps between stimulus blocks. In the silence condition, the ISI between stimuli was longer in the 2nd block than in the 1st and 3rd block.

The current experiment is similar to the classical oddball paradigm in that the same stimulus is presented as oddball in one block and as standard in the other block(s). However, unlike the classical oddball paradigm, in which the same stimulus is presented with different probabilities as oddball (low) and standard (high), in the current experiment, the target stimulus is presented with the same probability as oddball and standard. In the 2nd block, the target stimuli were presented as the oddball (deviant). In the 1st (only later 20 trials) and 3rd block, the target stimuli were presented as standard (do not contrast with other context stimuli). In the control condition, nothing changed across the 3 stimulus blocks, thus the deviant and standard are expected to elicit similar neural responses. In the diffZF condition, the context stimuli in the 2nd block were novel compared with those in the 1st and 3rd block. Thus, the familiar target stimulus is a deviant presented among 7 novel stimuli, and the deviant may be expected to elicit stronger neural responses than the standard. In the canary condition, the canary context stimuli and zebra finch target stimulus differed greatly in their acoustic features, e.g., pitch, pitch goodness, and entropy, as measured using Sound Analysis Pro (Tchernichovski et al., 2000) (Figure 2). Thus, the familiar target stimulus is a deviant presented among 7 context stimuli that are both novel and potentially of different acoustic category, and the deviant may be expected to elicit stronger neural responses than the standard. Because of the contrast both from familiarity and acoustic features, the response enhancement may be bigger than in the diffZF condition. In the silence condition, the average interval between sounds is much longer and the onset of a sound (the target stimulus) was much less predictable in the 2nd block than in the 1st and 3rd blocks. Thus, the deviant may be expected to elicit stronger neural responses than the standard.

Each experimental condition included two different stimulus sets (8 stimulus sets in total for 4 conditions) to control for potential stimulus-specific effects. The presentation order of these stimulus sets was counterbalanced across birds. Note that the first 20 trials of the target stimulus in the 1st block were excluded from analyses because neural responses show dynamic stimulus-specific adaptation in the zebra finch auditory forebrain (Chew et al., 1995, 1996).

### 2.7. Criterion for responsive electrodes and units

Recording sites (multi-unit) and units (single-unit) were included for data analysis if they responded to at least one deviant stimulus. Any given recording site or single-unit was considered to be responsive to a stimulus if these conditions were met:

1. The firing rate during the stimulus period was significantly different from that during the baseline period based on the paired Wilcoxon test (*p* < 0.001 for multi-unit, *p* < 0.05 for single-unit).
2. The average neural response to the stimulus was above baseline (firing rate > 30 spikes/s for multi-unit, > 3 spikes/s for single-unit). Consequently, only excitatory recording sites and units were included in the analyses.

### 2.8. Quantify neural response differences elicited by the deviant and the standard

The stimulus-specific adaptation index (SI) from Ulanovsky et al. (2003) was used to quantify the neural response differences elicited by the deviant and standard.

(1) $$\begin{array}{r} SI=\frac{R_{d}-R_{s}}{R_{d}+R_{s}} \end{array}$$

In the alternating oddball experiment, *R*_*d*_ and *R*_*s*_ represent the average neural responses to two stimuli when they were deviant and when they were standard. If the electrode/unit responded only to one of the stimuli, SI was calculated using the neural responses from the effective stimulus.

In the context oddball experiment, *R*_*d*_ and *R*_*s*_ represent the average neural response to the target stimulus when it was the deviant (in the 2nd block) and when it was the standard (in the 1st and 3rd block). Because each experimental condition included two different stimulus sets (see context oddball experiment), the averaged SI was used for statistical analyses whenever available (if the electrode responded to the target stimuli in both stimulus sets) for MUA. For SUA, because spike-sorting was performed separately for each stimulus set and the units from different stimulus sets cannot be guaranteed to be the same, SI from the same condition but different stimulus sets were pooled together for further statistical analyses.

### 2.9. Decoding with linear discriminant analysis (LDA)

Analyses of SI differences allow us to determine whether the deviant and standard stimuli elicited different neural responses. To explore whether these neural responses can be used by downstream neurons to decode whether responses were elicited by the deviant or the standard, LDA was employed using the following assumptions:

- For each bird, the population neural response to a stimulus trial is defined as the responses (firing rates) from all responsive electrodes/units. $\vec{x}=\left[x_{1},x_{2},\ldots ,x_{i},\ldots ,x_{n}\right]$, *n* is the total number of responsive electrodes/units.
- The population neural response to a stimulus is a random variable following a multivariate normal distribution. $\vec{x}\sim \text{MVN}\left(\vec{\mu },\Sigma \right)$.
- The population responses to the deviant and the standard have the same covariance matrix but different means.

Even though these assumptions may not be biologically plausible, they're consistent with the view that neural responses are samples from some underlying distributions (Hoyer and Hyvärinen, 2003; Buesing et al., 2011). The LDA classifier was used because it requires a relatively small sample size to train and is equivalent to the naive Bayes classifier when shrinkage parameter is set to 1. The used LDA is the implementation from the sklearn package in Python (Pedregosa et al., 2011).

In the alternating oddball experiment, each of the two stimuli was presented as the deviant for 20 trials and as the standard for 20 trials. For each stimulus, the LDA classifier was trained with 18 randomly sampled trials from the deviant and 18 trials from the standard, with the population responses as the features and deviant/standard as the labels. The trained LDA was then used to predict (classify) the labels of the 4 previously left-out trials and the percentage of correctly classified trials was stored. This procedure was repeated 500 times and the average percentage of correctly classified trials (classification accuracy) was calculated for each of the two stimuli. Their average (if available) was the decoding accuracy for a bird in the corresponding experimental condition.

In the context oddball experiment, there were 20 trials of population responses to the target stimulus in each of the three stimulus blocks (the first 20 trials in the 1st block were excluded). We first randomly sampled 19 trials from each of the 1st and 3rd block, and then randomly chose 18 trials as the population responses to the standard (to balance the number of samples from each class when training the LDA classifier); from the 2nd block, we randomly sampled 18 trials as the population responses to the deviant. The LDA was then trained on these data and used to predict (classify) the labels of the 4 previously left-out trials. This procedure was repeated 500 times and the average percentage of correctly classified trials (classification accuracy) was calculated. Since each experimental condition included two different stimulus sets, the decoding accuracy for a bird was the average classification accuracy from the two stimulus sets (if available).

### 2.10. Classifying units into narrow and wide type based on spike shape

Several recent studies suggested there may be two different types of neurons in the songbird auditory forebrain (Meliza and Margoliash, 2012; Menardy et al., 2012; Schneider and Woolley, 2013; Ono et al., 2016). One type had narrow spike waveforms and the other had wide spike waveforms. Single-units were classified into the wide and narrow types using the following method:

1. Calculate the average spike waveform for each unit and normalize the waveform by dividing it by its maximum.
2. Perform principal component analysis (PCA) for all the normalized spike waveforms and keep the first 4 components (explained more than 95% of all variances).
3. Use K-means clustering algorithm to classify the waveforms into two classes with the 4 components as features.
4. The units with wider average waveform are called wide and the rest are called narrow.

### 2.11. Histology

At the end of each recording experiment, several electric lesions (20 uA, 10 s) were made for later histological verification of the recording sites and the bird was returned to its home cage. Two days later, the bird was sacrificed with an overdose of pentobarbital (390 mg/ml, 2 ml), and perfused with 0.9% saline and 3.3% paraformaldehyde. After several days of fixation, the brain was cut into 50 um sagittal sections using a Vibrotome and stained with Cresyl Violet. Finally, the stained sections were visualized with a microscope and the anatomical positions of recording sites were reconstructed with respect to the previously made lesions. Figure 1 shows one example electrical lesion in the songbird auditory forebrain, which consists of field L2, NCM, and CM. Because the three structures did not show any systematic differences in the SI or decoding results, the data from them were pooled and reported together in both the alternating and context oddball experiments.

### 2.12. Statistical analyses

In the encoding analysis with SI, each subject is one electrode/unit. In the decoding analysis with LDA, each subject is a bird. For within-subject comparisons, we used paired sample *t*-test; for between-subject comparisons, we used independent sample *t*-test; for comparisons with hypothesized population means, we used one sample *t*-test. When the normality assumption was not met, corresponding non-parametric statistical tests (Wilcoxon test and Mann Whitney *U*-test) were used. In the decoding analysis, comparisons are within the same group of birds and thus we performed within-subject statistical tests for both MUA and SUA data. In the SI analysis, different statistical tests were used for comparisons based on MUA and SUA data. For MUA, comparisons are mostly within the same group of electrodes and thus we performed within-subject comparisons; for SUA, comparisons are across potentially different groups of units and thus we performed between-subject comparisons because spike sorting was done separately for different experimental conditions and units from different conditions cannot be guaranteed to be the same. The significance level was set at 0.05 (Bonferroni adjusted *p*-values were reported in the context oddball analysis using SI as 7 comparisons were conducted). All analyses were conducted using customized scripts in Spike2, Matlab, and Python.

In the alternating oddball experiment, control, alternating, and oddball condition had 97, 100, and 99 responsive electrodes (MUA), and 91, 72, and 83 responsive single-units (SUA). For the decoding analysis using LDA, all experimental conditions had the same 8 birds for both the MUA and SUA.

In the context oddball experiment, control, diffZF, canary, and silence condition had 89, 91, 90, and 91 responsive electrodes (MUA), and 77, 92, 94, and 98 responsive single-units (SUA). For the decoding analysis using LDA, all experimental conditions had the same 6 birds for both MUA and SUA. However, for SUA, one bird was excluded in the control and diffZF condition because not enough single-units were separated.

## 3. Results

### 3.1. Stimulus sequence influences neural responses to a stimulus

**Figure 5:**
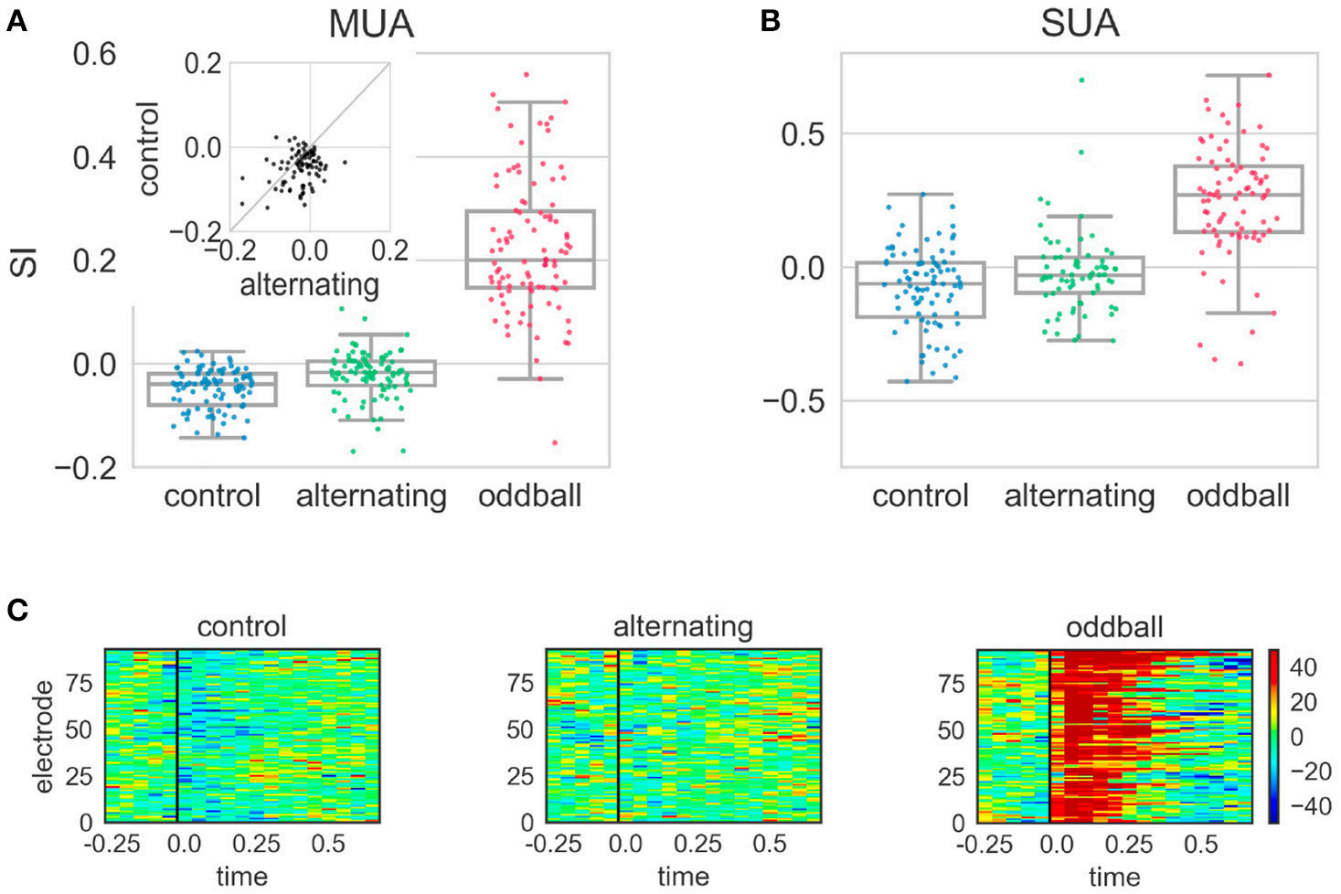
Stimulus-specific adaptation index (SI) and peri-stimulus time histogram (PSTH) differences in the alternating oddball experiment. **(A)** The SI for the multi-unit activity (MUA) in the three experimental conditions. Each dot represents the SI from one MUA site. Each box shows the quartiles of the dataset while the whiskers extend to the rest of the distribution, except for the potential outliers. The SI in the control condition was significantly smaller than 0. The SI in the alternating condition was significantly larger than in the control condition. The inset shows the paired comparison between the SI in the alternating and control conditions. Most dots are below the diagonal line at 45 degrees, showing the SI in the alternating condition was larger than in the control condition. **(B)** The SI from single unit activity showed similar results as MUA. **(C)** The PSTH differences (calculated for 50 ms bins from MUA) in different experimental conditions. Each panel indicates one experimental condition and each row indicates the color-coded amplitude of the PSTH difference from one electrode. The PSTH difference was the PSTH of the deviant minus the PSTH of the standard. The black line indicates the stimulus onset. In the control condition, the blue color after stimulus onset shows that the neural responses to the 2nd stimulus of the repeated pair were smaller than to the 1st stimulus. In the alternating condition, the homogeneous color shows that the PSTHs of the two stimuli of the repeated pair were similar. In the typical oddball condition, the dark red color shows that the neural responses to the deviant were much stronger than to the standard. Color scale indicates difference in spikes per second.

The different conditions tested in the alternating experiment produced distinct effects. In the control condition, the SI was significantly smaller than 0 [*t*_(96)_ = −8.31, *p* < 0.001], showing that the neural response to the 2nd stimulus of the repeated pair (repetition) was smaller than that to the 1st stimulus (Figure 5). Because the stimulus sequence was random and the 2nd stimulus in the repetition did not violate any regularities, this suggested neural responses to the 2nd stimulus of the repeated pair were suppressed following the 1st stimulus in the control condition. In contrast, the SI in the alternating condition was significantly larger than in the control condition [*t*_(93)_ = 3.17, *p* = 0.002], although it was still significantly smaller than 0 [*t*_(99)_ = −4.60, *p* < 0.001]. This showed that the suppressive effect was smaller when the repetition violated regularities in the stimulus sequence (Figure 5). Finally, the SI in the oddball condition was significantly larger than 0 and those in the control and alternating condition (*p* < 0.001 for all three comparisons). This oddball effect is consistent with previous reports (Ulanovsky et al., 2003; Beckers and Gahr, 2012).

Single-unit activity showed similar results (Figure 5) as the multi-unit activity. The SI in the control condition was significantly smaller than 0 [Wilcoxon *Z*_(90)_ = 1000, *p* < 0.001]. The SI in the alternating condition was larger than in the control condition, even though it was not statistically significant [Mann-Whitney *U*_(90, 71)_ = 2683, *p* = 0.024]. The SI in the alternating condition was not statistically different from 0 but showed a trend [Wilcoxon *Z*_(71)_ = 917, *p* = 0.026]. The SI in the oddball condition was significantly larger than 0 and those in the alternating and control condition (*p* < 0.001 for all three comparisons). Separate analyses were conducted on single-units that had been clustered into wide and narrow types based on their spike waveforms. Narrow spiking neurons are putative inhibitory whereas wide spiking neurons are putative excitatory, as suggested by several studies (Vates et al., 1996; Atencio and Schreiner, 2008; Schneider and Woolley, 2013). Consistent with these, in the current study, the narrow spiking neurons had significantly higher firing rates in response to zebra finch syllables than wide spiking neurons [*t*_(112, 89)_ = 3.32, *p* = 0.001]. However, the SIs of the wide and narrow spiking neurons were not significantly different from each other in either control or alternating conditions (*p* > 0.05 for both conditions).

### 3.2. Neural responses to stimuli in the repeated pair were harder to separate in the alternating condition than in the control condition

**Figure 6:**
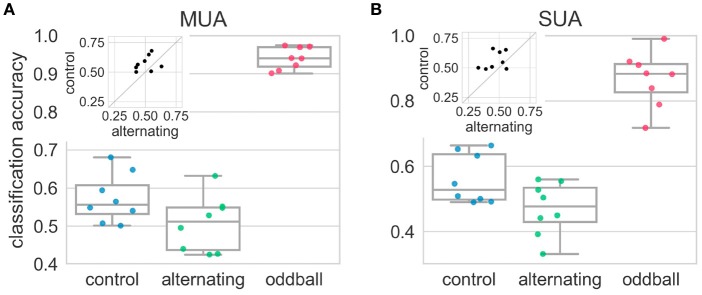
Decoding accuracies in the alternating oddball experiment for both multi-unit **(A)** and single-unit **(B)** activity. Each dot represents the decoding accuracy in one bird. The decoding accuracy in the alternating condition was not different from chance level (50%). The decoding accuracy in the control condition was much higher than in the alternating condition. The insets show paired comparisons between the alternating and control conditions, and most dots are above the diagonal line at 45°. The decoding accuracy in the typical oddball condition was much higher than chance level and higher than in the control and alternating conditions.

The results above showed that, at the neuronal level, the average neural responses elicited by the 2nd stimulus in the repetition (deviant) were more similar to those elicited by the 1st stimulus (standard) in the alternating condition than in the control condition. However, this result may be caused by the heterogeneity of the recorded sites/neurons: some sites/neurons may encode the deviant by responding more strongly but others may encode by decreasing their responses. This type of differences will be averaged out in the above analyses using SI. The LDA classification approach (see section 2.9) can find the dimensions that maximally separate the neural responses from two different distributions. Therefore, in any experimental condition, if the population responses to the 2nd and 1st stimulus really came from two different distributions, the decoding accuracy from the LDA should be significantly above the chance level (50% for two-classes classification). In the control condition, the decoding accuracy was significantly higher than chance level [*t*_(7)_ = 3.04, *p* = 0.02] (Figure 6). This suggested population responses can be used to decode whether population responses came from the 1st or 2nd stimulus in the repetition. However, in the alternating condition, the decoding accuracy was not different from chance level [*t*_(7)_ = 0.08, *p* = 0.94] and was much smaller than that in the control condition [*t*_(7)_ = −2.31, *p* = 0.054], suggesting population responses (using firing rates alone) cannot be used to decode the relative position of the stimulus in the repetition (Figure 6). In contrast, the average decoding accuracy in the oddball condition was around 90%, which was significantly higher than chancel level and those in the alternating and control condition (*p* < 0.01 for all three comparisons).

The results from single-unit activity were similar to those from the multi-unit activity. In the alternating condition, the decoding accuracy was at chance level [*t*_(7)_ = −1.06, *p* = 0.33] and was significantly smaller than that in the control condition [*t*_(7)_ = −2.33, *p* < 0.05] (Figure 6). The decoding accuracy in the control condition was much higher than the chancel level, even though it was not statistically significant [*t*_(7)_ = 2.27, *p* = 0.06]. This may be because some recording sessions had really few isolated single-units and not enough information was contained in these units. The average decoding accuracy from the oddball condition was around 90% and was significantly higher than chance level and those in the alternating and control condition (*p* < 0.01 for all three comparisons).

These decoding results, taken together with lack of SI differences in the wide and narrow neurons (clustered based on spike waveforms), ruled out the possibility that the heterogeneity of neurons caused the SI to be close to 0 in the alternating condition. This showed that neural responses to the 2nd and 1st stimulus of the repeated pair were more difficult to separate in the alternating condition than in the control condition. This raises the question how downstream neurons decode whether the observed neural responses came from the deviant or standard in the alternating condition.

### 3.3. Context affects neural responses to a stimulus

**Figure 7:**
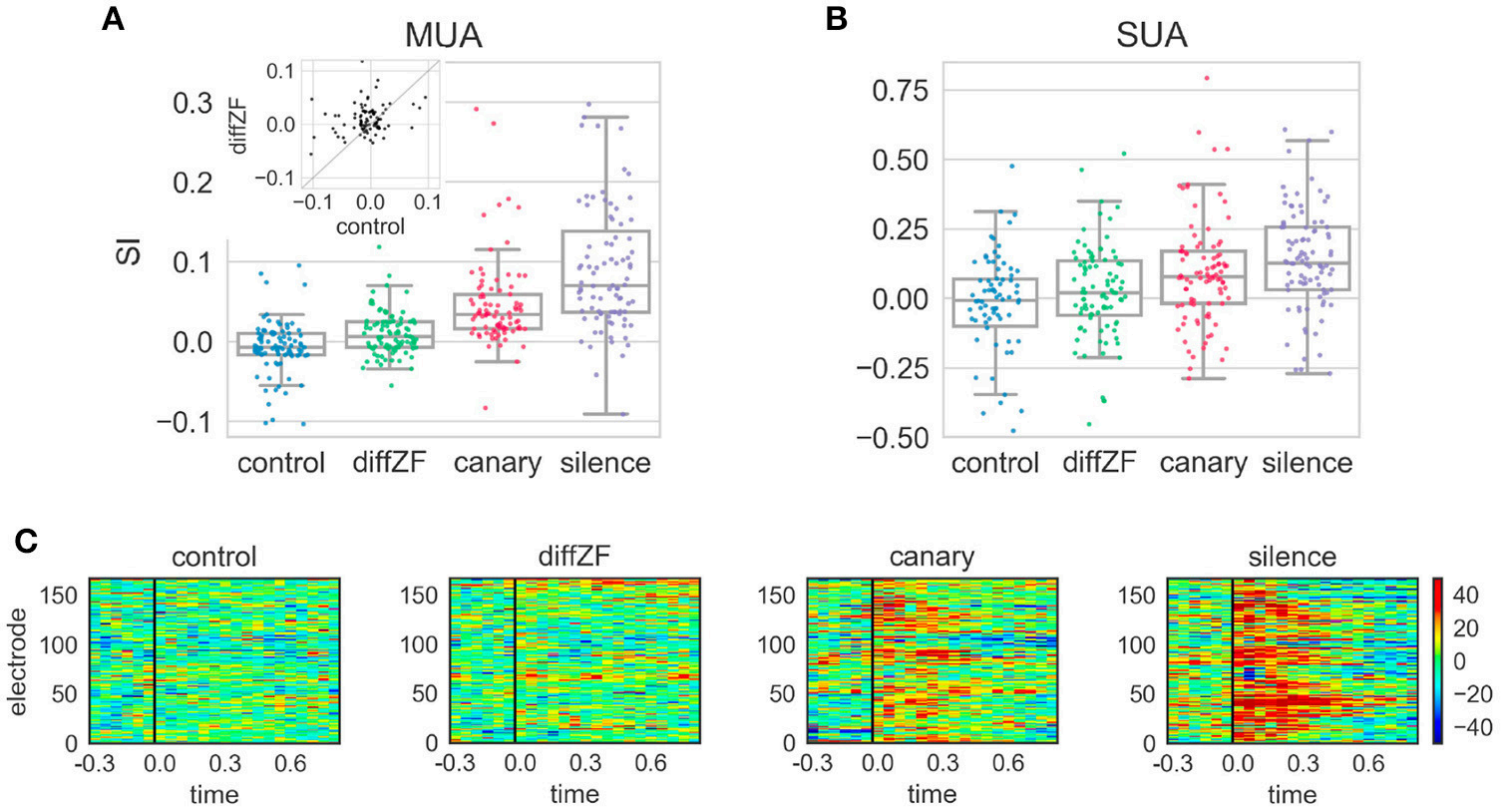
SI and PSTH difference in the context oddball experiment. **(A)** The SI from the four different experimental conditions. Each dot represents SI from one MUA site. The SI in the control condition was not different from 0. The SI in the diffZF, canary, and silence conditions each became larger, respectively, and all were significantly larger than in the control condition. The inset shows the paired comparison between the SI in the control and diffZF condition. Most of the dots are above the diagonal line at 45°, showing the SI in the diffZF condition was larger than in the control condition. **(B)** The SI from the single-unit activity (SUA) showed similar results as MUA. **(C)** The PSTH differences in the control, diffZF, canary, and silence conditions. Each panel shows one experimental condition and each row indicates the PSTH difference (calculated for 50 ms bins of the MUA) from one electrode. Conventions as in Figure 5. Note that each electrode occurs twice because each experimental condition included two different stimulus sets.

In the context oddball experiment, the response differences between the deviant and standard differed in different conditions. A one-way repeated measures ANOVA showed that context stimuli had significant effects on SI [*F*_(3, 261)_ = 55.420, *p* < 0.001]. *Post-hoc* analyses showed that the SI in the control condition was not different from 0 [*t*_(88)_ = −2.01, Bonferroni adjusted *p* = 0.18] (Figure 7). In the diffZF condition, the SI was significantly larger than in the control condition [*t*_(88)_ = 4.25, Bonferroni adjusted *p* < 0.001]. The SI in the diffZF condition was also significantly larger than 0 [*t*_(90)_ = 3.29, Bonferroni adjusted *p* = 0.003], even though the magnitude was small (mean = 0.01). This showed that the target stimulus elicited significantly larger neural responses during the 2nd block than during the 1st and 3rd block. The SI in the canary condition was significantly larger than in the diffZF condition [*t*_(88)_ = 4.36, Bonferroni adjusted *p* < 0.001]. Finally, the SI in the silence condition was significantly larger than in the canary condition [*t*_(89)_ = 3.98, Bonferroni adjusted *p* < 0.001] (Figure 7). These results showed that target stimulus elicited stronger neural responses during the 2nd block than during the 1st and 3rd block when the context stimuli changed across stimulus blocks. From diffZF, to canary to silence, the response enhancement became stronger and stronger.

Single-unit activity (Figure 7) showed similar results as the multi-unit activity. A one-way between-subjects ANOVA showed that context stimuli had significant effects on SI [*F*_(3, 358)_ = 5.619, *p* < 0.001]. In the control condition, the SI was not different from 0 [*t*_(76)_ = −2.2, Bonferroni adjusted *p* = 0.1] (Figure 7). In the diffZF condition, the SI was larger than in the control condition, even though this was not statistically significant [*t*_(91, 76)_ = 2.05, Bonferroni adjusted *p* = 0.05]. The SI in the canary and silence conditions were significantly larger than in the control condition (Bonferroni adjusted *p* < 0.01 for both comparisons). From diffZF, to canary to silence, the average SI increased gradually as in the multi-unit activity, even though these increases were not statistically significant (Bonferroni adjusted *p* > 0.05 for both comparisons).

### 3.4. A simple linear decoder is enough to decode whether a stimulus is oddball or standard

**Figure 8:**
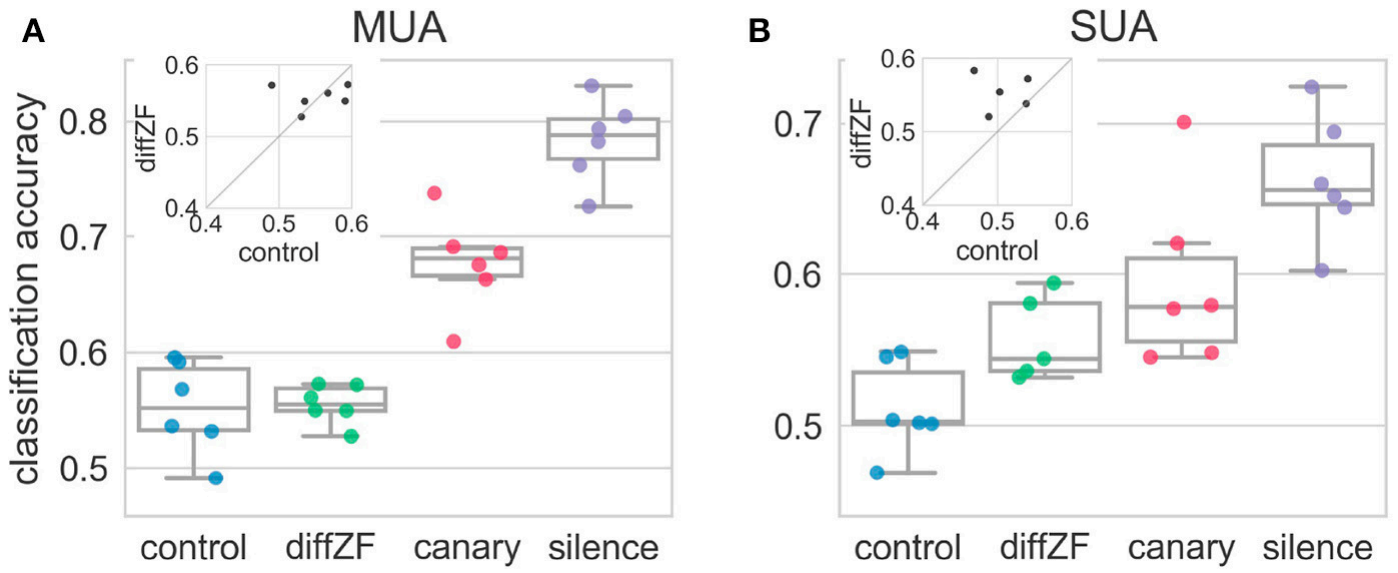
Decoding accuracies in the context oddball experiment for both multi-unit **(A)** and single-unit **(B)** activity. Each dot indicates decoding accuracy in one bird. The decoding accuracy in the control condition was not different from chance level. In contrast, the decoding accuracies from the diffZF, canary, and silence conditions became higher and higher, and were all significantly higher than chancel level. The insets show the paired comparison between the decoding accuracy in the control and the diffZF condition. The SUA data **(B)** show that the decoding accuracy in the diffZF condition was higher than in the control condition, whereas the MUA **(A)** do not.

LDA classification analysis (see section 2) was employed to test how downstream neurons may be using the neural responses to decode whether the stimulus responsible for the neural responses was the deviant or standard. A one-way repeated measures ANOVA showed that decoding accuracies were significantly different in different conditions [*F*_(3, 15)_ = 84.83, *p* < 0.001]. The decoding accuracy in the control condition was not different from chance level [*t*_(5)_ = 2.31, *p* > 0.05] (Figure 8). This was expected because the target stimulus was not an oddball in the control condition. In contrast, the classification accuracies in the diffZF, canary, and silence condition were all significantly higher than chance level (*p* < 0.05 for all three comparisons), and the accuracies became higher and higher (*p* < 0.01 for all consecutive comparisons). However, the decoding accuracy in the diffZF condition was not significantly different from the control condition [*t*_(5)_ = 0.29, *p* > 0.05] (Figure 8), potentially due to the small sample size.

Single-unit activity showed the same results as the multi-unit activity (Figure 8). A one-way between-subjects ANOVA showed that decoding accuracies in different conditions were significantly different [*F*_(3, 19)_ = 11.01, *p* < 0.001]. The decoding accuracy in the control condition was not different from chance level [*t*_(5)_ = 1.36, *p* > 0.05]. The decoding accuracies in the diffZF, canary, and silence were all significantly higher than chance level (*p* < 0.05 for all three comparisons). The decoding accuracy in the diffZF and canary condition were much higher than in the control condition. However, these were not statistically significant (*p* > 0.05, for both comparisons), potentially due to the small sample size. The decoding accuracy in the silence condition was significantly higher than in the control condition [*t*_(5)_ = 6.93, *p* < 0.01].

These results together showed that a simple linear decoder is enough to decode whether the neural responses was from the deviant or the standard in the context oddball experiment. However, the decoding accuracies differed in different experimental conditions, suggesting downstream neurons may need different neural mechanisms or different number of neurons to decode deviants in different experimental conditions.

## 4. Discussion

We investigated how the auditory system encoded and decoded deviant stimuli using both the alternating oddball and context oddball experiments. The alternating oddball experiment showed that neurons in the auditory forebrain were sensitive to the stimulus sequence, specifically the transition probabilities between stimuli. Responses to the deviant appear to reflect some combination of the current stimulus, the preceding stimulus, and the recent pattern of stimulus presentation. The context oddball experiment showed that the oddball effect can be elicited even when multiple standards were presented, as long as the standards are more similar to each other than to the target stimulus. The results using LDA showed that the difficult of decoding the stimulus responsible for the observed responses varied in different experimental conditions.

### 4.1. Repetition suppresses neural responses whereas deviance increases neural responses

In the typical oddball experiment, like that of Ulanovsky et al. (2003), the differences in the neural responses to the oddball and deviant were difficult to interpret. The difference could be because the neural responses to the repeated standard were smaller, or because the neural responses to the oddball were larger. In the control condition of the alternating oddball experiment, repetition occurred randomly in the stimulus sequence. Therefore, there was no specific expectation for the 2nd stimulus in the repetition; it was neither expected nor unexpected. However, the experimental results showed the 2nd stimulus in the repetition elicited significantly smaller neural responses than the 1st stimulus. Also, in this condition, the LDA analysis showed that the differences in population neural responses can be effectively used to decode whether the observed neural responses came from the 1st or 2nd stimulus in the repetition. These results together suggested repetition normally has a suppressive effect on the neural responses to the 2nd stimulus. Because the 2nd stimulus was neither expected nor unexpected, the suppressive effect cannot be easily explained by the predictive coding hypothesis (Rao and Ballard, 1999; Friston, 2005; Grotheer and Kovács, 2016; Heilbron and Chait, 2017). Instead, because the spectral-temporal structure of syllables was less complex than that of the songs and canary syllables often were just like complex tone sweeps (Figure 2), the suppressive effect may be more similar to forward masking or two-tone suppression (Ruggero et al., 1992; Alves-Pinto et al., 2010) and have a more mechanistic explanation. Past results have shown that forward masking can last from 50 to 500 ms (Brosch and Schreiner, 1997) and has been speculated to exist for ISIs longer than 500 ms (Wehr and Zador, 2005). However, the observed repetition suppression could also be due to other unknown mechanisms because repetition suppression has also been observed with much longer ISIs (≥ 7 s) in songbirds (Chew et al., 1995, 1996). This is consistent with the results from Todorovic and de Lange (2012) that showed repetition suppression may be separate from expectation suppression. Note that the current results showed that repetition suppression can happen in the absence of expectation but did not rule out the possibility that predictive coding can explain repetition suppression in certain conditions.

In contrast to the control condition, stimuli in the alternating condition were regularly alternating rather than randomly shuffled. Therefore, repetitions were unexpected and deviant. The results showed that the suppressive effect from repetition was significantly smaller in the alternating condition than in the control condition, implying the unexpectedness counteracted the repetition suppression and increased the neural responses to the 2nd stimulus. This suggests neurons in the songbird auditory forebrain learned the transition probabilities between stimuli and used them to predict the future stimulus. When the actual stimulus deviated from the prediction, the neural responses became stronger than what they normally would be. These results also suggested that both repetition suppression and prediction errors may be contributing to the oddball effect observed in the typical oddball experiments (Ulanovsky et al., 2003; Beckers and Gahr, 2010, 2012).

- Repetition suppresses the neural responses to the standard. The underlying mechanism can be predictive coding, or more mechanistic mechanisms as those in the forward masking, or both.
- Violation of prediction increases the neural responses to the oddball.

Parras et al. (2017) showed similar results about repetition suppression and prediction error using a different experiment paradigm adapted from Ruhnau et al. (2012). Our results are not simple replications. In the current experiment, alternation is the regular pattern and repetition is the deviant whereas it is the opposite in Parras et al. (2017). Our experimental results also showed that auditory neurons are sensitive to transition patterns between stimuli.

### 4.2. Neurons in the songbird auditory forebrain are sensitive to the transition probabilities between stimuli

The predictive coding hypothesis states that the sensory system is constantly improving its predictions about the future stimuli to reduce uncertainties in the environment (Friston, 2010). Prediction in the auditory domain requires the neurons to learn the transition patterns between stimuli and use it to predict the future stimuli. For the oddball effect in the typical oddball experiment, the simplest computational explanation is that the stimulus is presented with low probability as an oddball and thus less predictable than when it is the standard (Horvath et al., 2001; Ulanovsky et al., 2003; Khouri and Nelken, 2015). This does not require neurons to learn the transition probabilities between stimuli in order to detect the oddball. However, in the alternating condition, the two stimuli were presented with equal probabilities, and therefore the neurons must have learned the transition probabilities between stimuli to detect the repeated stimulus as a deviant. In addition, because the local sequence in the triplet (deviant, standard, and the stimulus before it) were identical in the alternating and control condition (Figure 3), the differences between alternating and control condition cannot be explained by synaptic habituation over the stimuli either (Ulanovsky et al., 2003; Mill et al., 2011a,b). The neural mechanisms underlying current results may be spike-timing dependent plasticity, which can theoretically implement a hidden-markov model (HMM) and learn temporal patterns between stimuli (Kappel et al., 2014, 2015). However, more experiments are needed to test this hypothesis.

For the experimental results in the alternating oddball experiment, an alternative explanation is that neurons may have treated “AB”/“BA” as the standard and “AA”/“BB” as the oddball. In the control condition, on average, “AA” and “BB” were presented 8 ± 3 times before the first test repetition (repetition in the triplet) was presented (≥ 5 repetitions each for 95% of the time). In contrast, there were no repetitions except the test repetitions in the alternating condition. If “AA”/“BB” had been treated as a composite stimuli by the birds, “AA”/“BB” would be more familiar to the birds and results from Chew et al. (1995), Chew et al. (1996), and Phan et al. (2006) would predict the adaptation to “AA”/“BB' is slower in the control condition than in the alternating condition. However, the adaptation rates for “AA” and “BB” were the same in the alternating and the control condition (Supplementary Figure 1). This suggests that it is unlikely that neurons have treated “AA”/“BB” as composite stimuli and thus supports the predictive coding hypothesis that neurons learned the transition probabilities between stimuli.

The time scale is the major difference between the current alternating experiment and other experiments that studied the effect of stimulus sequence on neural responses (Gill et al., 2008; Yaron et al., 2012; Schneider and Woolley, 2013; Lu and Vicario, 2014; Ono et al., 2016). Gill et al. (2008) showed the neural responses in caudal lateral mesopallium (CLM) of zebra finches can be better explained if the deviations from the local recent stimulus history are considered. This effect is limited to the time scale of the spectro-temporal receptive field (10 ms, STRF). Lu and Vicario (2014) showed that neurons in NCM can learn the transition probabilities between stimuli by taking advantage of the stimulus-specific adaptation (SSA) phenomenon found in the songbird (Chew et al., 1995, 1996), but there was no gaps between syllables in those experiments. Ono et al. (2016) used an oddball paradigm with complex stimuli consisting of several components and showed that neurons in the songbird were sensitive to the order between stimulus components. However, the interval between components of a complex stimulus is around 50 ms, which is much shorter than that in the current experiment. To some extent, differences in spectro-temporal receptive field (STRF) may be used to explain the results from the studies using short interval between stimuli (Ono et al., 2016), but this cannot be used to explain the current results which used a 1-second ISI. Even if the results from Ono et al. (2016) were not due to STRF difference, the bird may treat the several components as a composite stimulus and the underlying neural mechanisms may be different from that in the current experiment. Yaron et al. (2012), using an ISI comparable to the current results, showed that the oddball effect depended on the stimulus sequence, but they used the typical oddball paradigm and did not directly show that violation of transition probabilities resulted in different neural responses as in the current experiment. Wacongne et al. (2012) proposed a spiking neural network that can detect the deviant in the alternating condition. However, the ISI in their simulation was 150 ms and they did not test their model with a control condition. Our results, together with those from Ono et al. (2016), Lu and Vicario (2014), and Gentner et al. (2006), showed that songbirds are sensitive to sound sequences at various ISIs. These sequence processing abilities may be similar to those found in humans and monkeys (Wilson et al., 2015; Milne et al., 2017) and potentially share similar underlying neural mechanisms.

### 4.3. Oddball effects exist even when stimuli are complex and there are multiple standard stimuli

In the natural environment, sounds are often complex and a deviant stimulus occurs among many different “standard” stimuli. The different standard stimuli are similar to each other in certain acoustic dimensions whereas the deviant is not. The contrast in these dimensions creates the so-called deviant and standard. By using multiple complex stimuli differing in the familiarity or category (in acoustic feature space), the diffZF and canary condition mimicked a more complex and natural situation for deviance detection than the typical oddball paradigm. In the 2nd block of the diffZF condition, the target stimulus may be an oddball because it is familiar whereas the 7 context stimuli were novel. In the canary condition, the target stimulus may be an oddball in the sense that the target is a syllable from zebra finch songs whereas the context stimuli were both novel and potentially from a different acoustic category (syllables from canary songs). In both the diffZF and canary conditions of the context oddball experiment, the target stimulus elicited stronger neural responses in the 2nd block than in the 1st and 3rd block. This response enhancement may be similar to the oddball effect in the typical oddball experiment (Ulanovsky et al., 2003; Beckers and Gahr, 2012; Yaron et al., 2012). In the silence condition, there were no context stimuli and the ISI between stimuli was much longer and less predictable in the 2nd block than in the 1st and 3rd block. Therefore, the target stimulus may be an oddball in the 2nd block and elicited stronger neural responses than in the 1st and 3rd block. These results together demonstrated that the oddball effect exists even when stimuli are complex and there are multiple standard stimuli. These results are also consistent with the idea that sound processing in the auditory system is contextually modulated in both spectral and temporal domain (Angeloni and Geffen, 2018). However, as in the typical oddball experiment (Ulanovsky et al., 2003; Beckers and Gahr, 2012), the current results do not differentiate whether the response enhancement was caused by the violation of prediction or a more mechanistic interpretation like synaptic habituation. Because zebra finch and canary syllables differed in their acoustic structures (Figure 2), the response enhancement observed in the canary and the silence condition might be due to synaptic habituation, although this is less likely in the canary condition because of the multiple standards used. In the diffZF condition, all stimuli were zebra finch syllables and thus the effects are more difficult to explain with the synaptic habituation hypothesis. This suggests that violation of prediction probably is at least part of the explanation for the observed response enhancements in the diffZF condition.

The uncertain interpretation might be resolved by conducting the same experiments but presenting the canary syllables first with other canary syllables and then with zebra finch syllables. If the response enhancement is due to violation of the expectation, the responses to the target stimulus will be stronger when presented with zebra finch syllables. If it is due to synaptic habituation, the responses to the target stimulus should be lower when presented with zebra finch syllables. Lu and Vicario (2017) did such an experiment and the results were opposite to the latter prediction, suggesting the response enhancement is not due to synaptic habituation. However, the ISI in Lu and Vicario (2017) was 7 seconds and it is not clear whether the results will be similar when a shorter ISI is used. In the canary and silence conditions, the current experimental results were similar to those of Lu and Vicario (2017), suggesting that deviance detection may be operating across very different time scales. Farley et al. (2010) did a similar experiment and showed that the oddball effect reflected the rarity rather than the deviance of the oddball stimulus. However, the results are difficult to compare because of three differences: type of stimuli used (pure tone vs. complex syllables), whether stimulus blocks were repeated (10 vs. 1), and the state of the subject (anesthetized vs. awake).

### 4.4. Decoding the neural responses from the deviant and the standard

The neural response differences elicited by the deviant and standard were quantified by SI. However, this measure does not tell us whether and how these responses may be used to decode the neural responses as being elicited by the deviant vs. the standard on a trial-by-trial basis. The results using LDA showed that the neural responses from the deviant and standard can be distinguished and decoded in the context oddball, typical oddball, and even the control condition of the alternating oddball experiment (all with above-chance level accuracy). However, in the alternating condition, the neural responses were indistinguishable and the LDA failed to decode whether the neural responses were from the deviant or standard. Note that this does not mean the brain cannot distinguish the deviant and the standard. The brain has access to more information (e.g., more neurons from the auditory forebrain and/or other auditory structures) and may also have more complex decoding mechanisms. In humans, at least, Dehaene et al. (2015), Nordby et al. (1988), and Näätänen et al. (1993) showed that the EEG/MEG responses from the deviant and standard are different in the alternating condition. In the end, the differences in the decoding results between the typical/context and the alternating oddball conditions, suggested that different neural mechanisms may be used to detect different types of oddballs (deviants).

Compared with the typical encoding analysis method using SI, the decoding analysis using LDA (or other decoding methods) has the following potential advantages:

- It directly tests whether the observed differences in neural responses between stimuli are large and consistent enough to enable decoding.
- It can detect the differences that measurements like SI cannot. For example, if the neurons were heterogeneous and some neurons increased their neural responses to the deviant whereas other decreased their responses, the average SI may not show a difference statistically. However, the decoding analysis can detect the effect as long as the differences were consistent within each neuron.
- It can be used to test whether the correlation between neurons may encode information. For example, the shrinkage parameter in the LDA controls how much covariance between different neurons were used for the classification and thus decoding. In the current experiment, similar results were obtained using different shrinkage values (data not shown), suggesting the correlation between neurons may be not useful in decoding the deviant and standard.

In summary, the current study shows that neurons in the songbird auditory forebrain are sensitive to the transition probabilities between stimuli. When the prediction based on the learned transition probabilities was violated, the neurons responded more strongly than they normally would. This provides new evidence for the predictive coding hypothesis and clarifies the neural mechanisms underlying the oddball effect in the typical oddball condition, which may include both an increased response to the oddball and a decreased response to the standard. The alternating and context oddball experiments together showed that more complex oddballs are also encoded in the auditory system, but the decoding difficulties differ. These two experiments also provide potential new ways to explore the neural correlate of MMN because they cannot be simply explained by synaptic habituation and the experiment paradigms are similar to those used in the MMN literature (Nordby et al., 1988; Sharma and Dorman, 2000; Cornella et al., 2012; Silva et al., 2017). Because our birds were not anesthetized and could attend to the auditory stream, our results may be related to P300, another correlate of prediction error that requires attention (Wacongne et al., 2011; Chennu et al., 2013). However, much more experiments have to be conducted before reaching any conclusion. In its simplest form, spoken language is a series of sounds produced based on certain transition probabilities between syllables and words. Auditory sequence processing have been suggested to be evolutionarily conservative in humans and non-human primates (Wilson et al., 2015). The current experiments showed that zebra finches also process sounds in a sequence-sensitive manner and may provide insights into the neural mechanisms of sequence sensitivity in speech processing.

## Author contributions

MD and DV conceived and designed research, interpreted results of experiments, drafted manuscript, edited and revised manuscript, and approved final version of manuscript. MD performed experiments, analyzed data, and prepared figures.

### Conflict of interest statement

The authors declare that the research was conducted in the absence of any commercial or financial relationships that could be construed as a potential conflict of interest.

## References

[B1] Alves-Pinto A. Baudoux S. Palmer A. R. Sumner C. J. (2010). Forward masking estimated by signal detection theory analysis of neuronal responses in primary auditory cortex. J. Assoc. Res. Otolaryngol. 11, 477–494. 10.1007/s10162-010-0215-6 20369270 PMC2914239

[B2] Angeloni C. Geffen M. (2018). Contextual modulation of sound processing in the auditory cortex. Curr. Opin. Neurobiol. 49, 8–15. 10.1016/j.conb.2017.10.012 29125987 PMC6037899

[B3] Antunes F. M. Malmierca M. S. (2011). Effect of auditory cortex deactivation on stimulus-specific adaptation in the medial geniculate body. J. Neurosci. 31, 17306–17316. 10.1523/JNEUROSCI.1915-11.2011 22114297 PMC6623836

[B4] Antunes F. M. Nelken I. Covey E. Malmierca M. S. (2010). Stimulus-specific adaptation in the auditory thalamus of the anesthetized rat. PLoS ONE 5:e14071. 10.1371/journal.pone.0014071 21124913 PMC2988819

[B5] Atencio C. A. Schreiner C. E. (2008). Spectrotemporal processing differences between auditory cortical fast-spiking and regular-spiking neurons. J. Neurosci. 28, 3897–3910. 10.1523/JNEUROSCI.5366-07.2008 18400888 PMC2474630

[B6] Beckers G. J. Gahr M. (2010). Neural processing of short-term recurrence in songbird vocal communication. PLoS ONE 5:e11129. 10.1371/journal.pone.0011129 20567499 PMC2886052

[B7] Beckers G. J. Gahr M. (2012). Large-scale synchronized activity during vocal deviance detection in the zebra finch auditory forebrain. J. Neurosci. 32, 10594–10608. 10.1523/JNEUROSCI.6045-11.2012 22855809 PMC6621405

[B8] Brainard M. S. Doupe A. J. (2013). Translating birdsong: songbirds as a model for basic and applied medical research. Annu. Rev. Neurosci. 36, 489–517. 10.1146/annurev-neuro-060909-152826 23750515 PMC4130661

[B9] Brosch M. Schreiner C. E. (1997). Time course of forward masking tuning curves in cat primary auditory cortex. J. Neurophysiol. 77, 923–943. 10.1152/jn.1997.77.2.923 9065859

[B10] Buesing L. Bill J. Nessler B. Maass W. (2011). Neural dynamics as sampling: a model for stochastic computation in recurrent networks of spiking neurons. PLoS Comput. Biol. 7:e1002211. 10.1371/journal.pcbi.1002211 22096452 PMC3207943

[B11] Chennu S. Noreika V. Gueorguiev D. Blenkmann A. Kochen S. Ibánez A.. (2013). Expectation and attention in hierarchical auditory prediction. J. Neurosci. 33, 11194–11205. 10.1523/JNEUROSCI.0114-13.2013 23825422 PMC3718380

[B12] Chew S. J. Mello C. Nottebohm F. Jarvis E. Vicario D. S. (1995). Decrements in auditory responses to a repeated conspecific song are long-lasting and require two periods of protein synthesis in the songbird forebrain. Proc. Natl. Acad. Sci. U.S.A. 92, 3406–3410. 10.1073/pnas.92.8.3406 7724575 PMC42175

[B13] Chew S. J. Vicario D. S. Nottebohm F. (1996). A large-capacity memory system that recognizes the calls and songs of individual birds. Proc. Natl. Acad. Sci. U.S.A. 93, 1950–1955. 10.1073/pnas.93.5.1950 8700865 PMC39889

[B14] Cornella M. Leung S. Grimm S. Escera C. (2012). Detection of simple and pattern regularity violations occurs at different levels of the auditory hierarchy. PLoS ONE 7:e43604. 10.1371/journal.pone.0043604 22916282 PMC3423368

[B15] Dehaene S. Meyniel F. Wacongne C. Wang L. Pallier C. (2015). The neural representation of sequences: from transition probabilities to algebraic patterns and linguistic trees. Neuron 88, 2–19. 10.1016/j.neuron.2015.09.019 26447569

[B16] Farley B. J. Quirk M. C. Doherty J. J. Christian E. P. (2010). Stimulus-specific adaptation in auditory cortex is an nmda-independent process distinct from the sensory novelty encoded by the mismatch negativity. J. Neurosci. 30, 16475–16484. 10.1523/JNEUROSCI.2793-10.2010 21147987 PMC6634869

[B17] Friston K. (2005). A theory of cortical responses. Philos. Trans. R. Soc. Lond. B Biol. Sci. 360, 815–836. 10.1098/rstb.2005.1622 15937014 PMC1569488

[B18] Friston K. (2010). The free-energy principle: a unified brain theory? Nat. Rev. Neurosci. 11, 127–138. 10.1038/nrn2787 20068583

[B19] Friston K. Kiebel S. (2009). Predictive coding under the free-energy principle. Philos. Trans. R. Soc. Lond. B Biol. Sci. 364, 1211–1221. 10.1098/rstb.2008.0300 19528002 PMC2666703

[B20] Garrido M. I. Kilner J. M. Stephan K. E. Friston K. J. (2009). The mismatch negativity: a review of underlying mechanisms. Clin. Neurophysiol. 120, 453–463. 10.1016/j.clinph.2008.11.029 19181570 PMC2671031

[B21] Gentner T. Q. Fenn K. M. Margoliash D. Nusbaum H. C. (2006). Recursive syntactic pattern learning by songbirds. Nature 440, 1204–1207. 10.1038/nature04675 16641998 PMC2653278

[B22] Gill P. Woolley S. M. Fremouw T. Theunissen F. E. (2008). Whats that sound? Auditory area clm encodes stimulus surprise, not intensity or intensity changes. J. Neurophysiol. 99, 2809–2820. 10.1152/jn.01270.2007 18287545

[B23] Grotheer M. Kovács G. (2016). Can predictive coding explain repetition suppression? Cortex 80, 113–124. 10.1016/j.cortex.2015.11.027 26861559

[B24] Heilbron M. Chait M. (2017). Great expectations: is there evidence for predictive coding in auditory cortex? Neuroscience 389, 54–73. 10.1016/j.neuroscience.2017.07.061 28782642

[B25] Hershenhoren I. Taaseh N. Antunes F. M. Nelken I. (2014). Intracellular correlates of stimulus-specific adaptation. J. Neurosci. 34, 3303–3319. 10.1523/JNEUROSCI.2166-13.2014 24573289 PMC6795306

[B26] Horvath J. Czigler I. Sussman E. Winkler I. (2001). Simultaneously active pre-attentive representations of local and global rules for sound sequences in the human brain. Cogn. Brain Res. 12, 131–144. 10.1016/S0926-6410(01)00038-6 11489616

[B27] Hoyer P. O. Hyvärinen A. (2003). Interpreting neural response variability as monte carlo sampling of the posterior, in Advances in Neural Information Processing Systems (Vancouver, BC), 293–300.

[B28] Kappel D. Habenschuss S. Legenstein R. Maass W. (2015). Network plasticity as bayesian inference. PLoS Comput. Biol. 11:e1004485. 10.1371/journal.pcbi.1004485 26545099 PMC4636322

[B29] Kappel D. Nessler B. Maass W. (2014). Stdp installs in winner-take-all circuits an online approximation to hidden markov model learning. PLoS Comput. Biol. 10:e1003511. 10.1371/journal.pcbi.1003511 24675787 PMC3967926

[B30] Khouri L. Nelken I. (2015). Detecting the unexpected. Curr. Opin. Neurobiol. 35, 142–147. 10.1016/j.conb.2015.08.003 26318534

[B31] Lu K. Vicario D. S. (2017). Familiar but unexpected: effects of sound context statistics on auditory responses in the songbird forebrain. J. Neurosci. 37, 12006–12017. 10.1523/JNEUROSCI.5722-12.2017 29118103 PMC5719976

[B32] Lu K. Vicario D. S. (2014). Statistical learning of recurring sound patterns encodes auditory objects in songbird forebrain. Proc. Natl. Acad. Sci. U.S.A. 111, 14553–14558. 10.1073/pnas.1412109111 25246563 PMC4210023

[B33] Malmierca M. S. Anderson L. A. Antunes F. M. (2015). The cortical modulation of stimulus-specific adaptation in the auditory midbrain and thalamus: a potential neuronal correlate for predictive coding. Front. Syst. Neurosci. 9:19. 10.3389/fnsys.2015.00019 25805974 PMC4353371

[B34] Meliza C. D. Margoliash D. (2012). Emergence of selectivity and tolerance in the avian auditory cortex. J. Neurosci. 32, 15158–15168. 10.1523/JNEUROSCI.0845-12.2012 23100437 PMC3498467

[B35] Menardy F. Touiki K. Dutrieux G. Bozon B. Vignal C. Mathevon N.. (2012). Social experience affects neuronal responses to male calls in adult female zebra finches. Eur. J. Neurosci. 35, 1322–1336. 10.1111/j.1460-9568.2012.08047.x 22512260

[B36] Mill R. Coath M. Wennekers T. Denham S. L. (2011a). Abstract stimulus-specific adaptation models. Neural Comput. 23, 435–476. 10.1162/NECO_a_00077 21114400

[B37] Mill R. Coath M. Wennekers T. Denham S. L. (2011b). A neurocomputational model of stimulus-specific adaptation to oddball and markov sequences. PLoS Comput. Biol. 7:e1002117. 10.1371/journal.pcbi.1002117 21876661 PMC3158038

[B38] Milne A. E. Petkov C. I. Wilson B. (2017). Auditory and visual sequence learning in humans and monkeys using an artificial grammar learning paradigm. Neuroscience 389, 104–117. 10.1016/j.neuroscience.2017.06.059 28687306 PMC6278909

[B39] Näätänen R. Paavilainen P. Rinne T. Alho K. (2007). The mismatch negativity (mmn) in basic research of central auditory processing: a review. Clin. Neurophysiol. 118, 2544–2590. 10.1016/j.clinph.2007.04.026 17931964

[B40] Näätänen R. Paavilainen P. Titinen H. Jiang D. Alho K. (1993). Attention and mismatch negativity. Psychophysiology 30, 436–450. 10.1111/j.1469-8986.1993.tb02067.x 8416070

[B41] Nieto-Diego J. Malmierca M. S. (2016). Topographic distribution of stimulus-specific adaptation across auditory cortical fields in the anesthetized rat. PLoS Biol. 14:e1002397. 10.1371/journal.pbio.1002397 26950883 PMC4780834

[B42] Nordby H. Roth W. T. Pfefferbaum A. (1988). Event-related potentials to breaks in sequences of alternating pitches or interstimulus intervals. Psychophysiology 25, 262–268. 10.1111/j.1469-8986.1988.tb01239.x 3406327

[B43] Ono S. Okanoya K. Seki Y. (2016). Hierarchical emergence of sequence sensitivity in the songbird auditory forebrain. J. Comparat. Physiol. A 202, 163–183. 10.1007/s00359-016-1070-7 26864094

[B44] Paavilainen P. (2013). The mismatch-negativity (mmn) component of the auditory event-related potential to violations of abstract regularities: a review. Int. J. Psychophysiol. 88, 109–123. 10.1016/j.ijpsycho.2013.03.015 23542165

[B45] Parras G. G. Nieto-Diego J. Carbajal G. V. Valdés-Baizabal C. Escera C. Malmierca M. S. (2017). Neurons along the auditory pathway exhibit a hierarchical organization of prediction error. Nat. Commun. 8:2148. 10.1038/s41467-017-02038-6 29247159 PMC5732270

[B46] Pedregosa F. Varoquaux G. Gramfort A. Michel V. Thirion B. Grisel O. (2011). Scikit-learn: machine learning in Python. J. Mach. Learn. Res. 12, 2825–2830. Available Online at: http://www.jmlr.org/papers/v12/pedregosa11a.html

[B47] Phan M. L. Pytte C. L. Vicario D. S. (2006). Early auditory experience generates long-lasting memories that may subserve vocal learning in songbirds. Proc. Natl. Acad. Sci. U.S.A. 103, 1088–1093. 10.1073/pnas.0510136103 16418265 PMC1327730

[B48] Quiroga R. Q. Nadasdy Z. Ben-Shaul Y. (2004). Unsupervised spike detection and sorting with wavelets and superparamagnetic clustering. Neural Comput. 16, 1661–1687. 10.1162/089976604774201631 15228749

[B49] Rao R. P. Ballard D. H. (1999). Predictive coding in the visual cortex: a functional interpretation of some extra-classical receptive-field effects. Nat. Neurosci. 2:79. 10.1038/4580 10195184

[B50] Ruggero M. A. Robles L. Rich N. C. (1992). Two-tone suppression in the basilar membrane of the cochlea: mechanical basis of auditory-nerve rate suppression. J. Neurophysiol. 68, 1087–1099. 10.1152/jn.1992.68.4.1087 1432070

[B51] Ruhnau P. Herrmann B. Schröger E. (2012). Finding the right control: the mismatch negativity under investigation. Clin. Neurophysiol. 123, 507–512. 10.1016/j.clinph.2011.07.035 21839676

[B52] Schneider D. M. Woolley S. M. (2013). Sparse and background-invariant coding of vocalizations in auditory scenes. Neuron 79, 141–152. 10.1016/j.neuron.2013.04.038 23849201 PMC3713513

[B53] Sharma A. Dorman M. F. (2000). Neurophysiologic correlates of cross-language phonetic perception. J. Acoust. Soc. Am. 107, 2697–2703. 10.1121/1.428655 10830391

[B54] Silva D. M. Melges D. B. Rothe-Neves R. (2017). N1 response attenuation and the mismatch negativity (mmn) to within-and across-category phonetic contrasts. Psychophysiology 54, 591–600. 10.1111/psyp.12824 28169421

[B55] Tchernichovski O. Nottebohm F. Ho C. E. Pesaran B. Mitra P. P. (2000). A procedure for an automated measurement of song similarity. Anim. Behav. 59, 1167–1176. 10.1006/anbe.1999.1416 10877896

[B56] Todorovic A. de Lange F. P. (2012). Repetition suppression and expectation suppression are dissociable in time in early auditory evoked fields. J. Neurosci. 32, 13389–13395. 10.1523/JNEUROSCI.2227-12.2012 23015429 PMC6621367

[B57] Ulanovsky N. Las L. Nelken I. (2003). Processing of low-probability sounds by cortical neurons. Nat. Neurosci. 6, 391–398. 10.1038/nn1032 12652303

[B58] Vates G. E. Broome B. M. Mello C. V. Nottebohm F. (1996). Auditory pathways of caudal telencephalon and their relation to the song system of adult male zebra finches (taenopygia guttata). J. Comparat. Neurol. 366, 613–642. 10.1002/(SICI)1096-9861(19960318)366:4<613::AID-CNE5>3.0.CO;2-7 8833113

[B59] Wacongne C. Changeux J.-P. Dehaene S. (2012). A neuronal model of predictive coding accounting for the mismatch negativity. J. Neurosci. 32, 3665–3678. 10.1523/JNEUROSCI.5003-11.2012 22423089 PMC6703454

[B60] Wacongne C. Labyt E. van Wassenhove V. Bekinschtein T. Naccache L. Dehaene S. (2011). Evidence for a hierarchy of predictions and prediction errors in human cortex. Proc. Natl. Acad. Sci. U.S.A. 108, 20754–20759. 10.1073/pnas.1117807108 22147913 PMC3251061

[B61] Wehr M. Zador A. M. (2005). Synaptic mechanisms of forward suppression in rat auditory cortex. Neuron 47, 437–445. 10.1016/j.neuron.2005.06.009 16055066

[B62] Wilson B. Kikuchi Y. Sun L. Hunter D. Dick F. Smith K.. (2015). Auditory sequence processing reveals evolutionarily conserved regions of frontal cortex in macaques and humans. Nat. Commun. 6:8901. 10.1038/ncomms9901 26573340 PMC4660034

[B63] Yaron A. Hershenhoren I. Nelken I. (2012). Sensitivity to complex statistical regularities in rat auditory cortex. Neuron 76, 603–615. 10.1016/j.neuron.2012.08.025 23141071

