# Supplementary material for: Neural Correlate of Transition Violation and Deviance Detection in the Songbird Auditory Forebrain
Source: Front Syst Neurosci. 2018 Oct 9;12:46. doi: 10.3389/fnsys.2018.00046 (PMC6190688; doi:10.3389/fnsys.2018.00046)
Supplement: Supplementary file 1 [file Table_1.DOCX]

Supplementary Material

Neural correlate of transition violation and deviance detection in the songbird auditory forebrain

Mingwen Dong*, David S. Vicario

*** Correspondence:** Corresponding Author: mingwen.dong@rutgers.edu

## Supplementary Figures


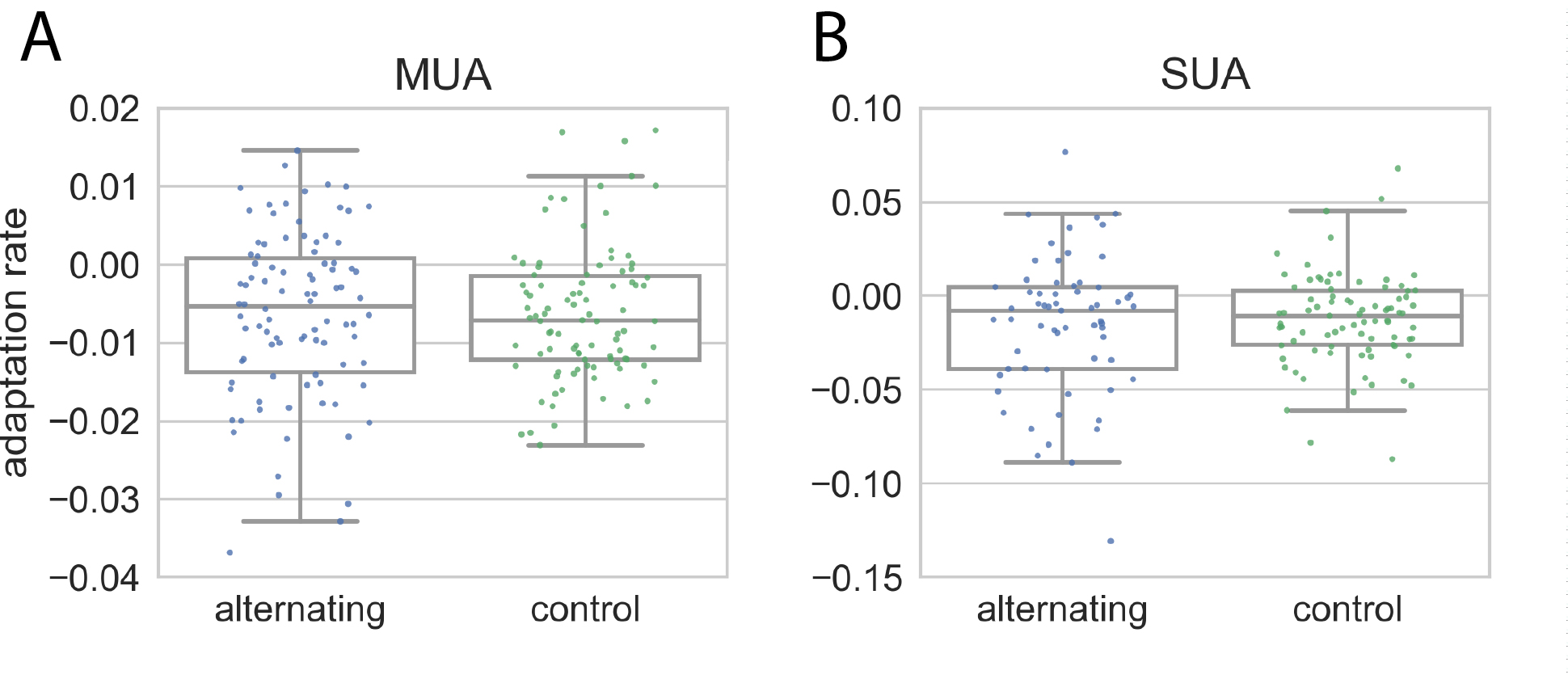


**Supplementary Figure 1.** Adaptation rates for “AA”/”BB” composite stimuli in the alternating oddball experiment (see Discussion). The adaptation rates in the alternating and control conditions were not different from each other (t(93) = -0.363, p = 0.71 for MUA; t(151) = -0.964, p = 0.336 for SUA). This suggests that, in the alternating condition, the response enhancement to the 2^nd^ stimulus in the repeated repair is unlikely due to the possibility that neurons have treated "AB"/"BA" as the standard and "AA"/"BB" as the oddball. This supports our conclusion that violation of alternation patterns in the preceding stimulus sequence increases neural responses to repeated stimuli. Adaptation (habituation) rate is calculated using the method described in Chew et al. [1996] and Phan et al. [2006].
